# A Bayesian data fusion based approach for learning genome-wide transcriptional regulatory networks

**DOI:** 10.1186/s12859-020-3510-1

**Published:** 2020-05-29

**Authors:** Elisabetta Sauta, Andrea Demartini, Francesca Vitali, Alberto Riva, Riccardo Bellazzi

**Affiliations:** 1 Department of Electrical, Computer and Biomedical Engineering, University of Pavia, Via Ferrata 5, 27100 Pavia, Italy; 2 Center for Biomedical Informatics and Biostatistics, Dept. of Medicine, The University of Arizona Health Sciences, 1230 Cherry Ave, Tucson, AZ 85719 USA; 3 Bioinformatics Core, Interdisciplinary Center for Biotechnology Research, University of Florida, Gainesville, FL 32610 USA

**Keywords:** Genomic transcriptional networks, *omics*-data fusion, Bayesian networks, Hybrid structure learning algorithm

## Abstract

**Background:**

Reverse engineering of transcriptional regulatory networks (TRN) from genomics data has always represented a computational challenge in System Biology. The major issue is modeling the complex crosstalk among transcription factors (TFs) and their target genes, with a method able to handle both the high number of interacting variables and the noise in the available heterogeneous experimental sources of information.

**Results:**

In this work, we propose a data fusion approach that exploits the integration of complementary *omics*-data as prior knowledge within a Bayesian framework, in order to learn and model large-scale transcriptional networks. We develop a hybrid structure-learning algorithm able to jointly combine TFs ChIP-Sequencing data and gene expression compendia to reconstruct TRNs in a genome-wide perspective. Applying our method to high-throughput data, we verified its ability to deal with the complexity of a genomic TRN, providing a snapshot of the synergistic TFs regulatory activity.

Given the noisy nature of data-driven prior knowledge, which potentially contains incorrect information, we also tested the method’s robustness to false priors on a benchmark dataset, comparing the proposed approach to other regulatory network reconstruction algorithms. We demonstrated the effectiveness of our framework by evaluating structural commonalities of our learned genomic network with other existing networks inferred by different DNA binding information-based methods.

**Conclusions:**

This Bayesian omics-data fusion based methodology allows to gain a genome-wide picture of the transcriptional interplay, helping to unravel key hierarchical transcriptional interactions, which could be subsequently investigated, and it represents a promising learning approach suitable for multi-layered genomic data integration, given its robustness to noisy sources and its tailored framework for handling high dimensional data.

## Background

The transcriptional regulatory machinery consists of cooperative interactions among transcription factors (TFs) responsible for regulating the spatial and temporal expression of genes in response to different cellular stimuli. Dysregulation of such transcriptional programs is one of the key hallmarks of cancer, affecting the clinical progression and the therapeutic responsiveness of the disease phenotype [1]. A promising approach for investigating the altered transcriptional response underlying cancer is to reconstruct the transcriptional dependencies among TFs and their target genes as a network, exploiting the genome-wide scale and the complementary data types offered by high-throughput technologies, to mine the resulting regulatory structure and extract interactions pattern from the genomic transcriptional hierarchy of the considered phenotype [2, 3].

Modelling such complex transcriptional regulatory networks (TRNs) represents one of the most challenging task in Computational Biology, given the high dimensionality of involved interactors and that their molecular dynamics are not fully understood [4]. For this reason, computational modeling is an essential component in reverse engineering of transcriptional networks. As He and Tan pointed out in their recent review [5], among current computational approaches for constructing TRNs, there is a lack of integrative genome-wide methods which combine *omics*-data sources to strengthen the accuracy of the obtained models and to provide novel insights from the inferred network structure. A particularly important issue is to find a method able to deal with the biological complexity of these systems, and that is sufficiently robust to scale their genomic dimension allowing multiple data integration.

During the last years, this aspect has been increasingly emphasized along with the rapid growth of high-throughput genomic data types. A variety of approaches have been exploited to predict the interaction of regulatory elements [6–8], including models focused on reconstructing physical locations of transcription factors through analysis of DNA sequence information, either using TFs binding site motifs, or chromatin accessibility data, as measured by DNase I hypersensitivity sites sequencing (DNase-Seq) or by transposase-accessible chromatin sequencing (ATAC-Seq) [7, 9–11]. Nevertheless, the regulatory activity that can be predicted from these binding affinities is limited to the set of TFs whose specific molecular sequences have been characterized, without taking into account the capability of certain TFs to recognize multiple motifs and the interaction with other cofactors, losing a proportion of potential transcriptional dependencies, that may help to depict a more comprehensive regulatory schema [12, 13].

Other computational strategies rely on learning the wiring transcriptional architecture, which orchestrates cellular gene expression, from transcriptomic data (in particular obtained by microarray experiments, widely available in different conditions and contexts) as a sole or primary source [14], using mathematical approaches including Boolean networks, information theoretic or correlation-based methods, differential equations systems, Bayesian and Neural networks [15–17]. Among these, Bayesian Networks (BNs) have become the prominent technique to model TRNs for their probabilistic formalism that can reflect the stochastic and combinatorial nature of gene regulation and for their ability to handle incomplete noisy data [18–20]. In this way, the network structure, constituted of causal and non-causal regulatory relationships among biological factors, is learned from genomic expression profiles, within a static or a dynamic schema. Friedman et al. [21] and Murphy and Mian [22] were among the first to apply a Bayesian structure learning strategy on time-series data, trying to capture transcriptional dynamics in the temporal domain. The limited number of monitored time points is nevertheless statistically insufficient for reconstructing even a moderately-sized network, making this approach not suitable for human genome-wide transcriptional networks. Other methods [20, 23, 24] have focused their learning procedure only on static gene expression profiles that could produce unreliable biological transcriptional regulations, due to the noisy nature of this experimental source. Moreover, learning networks from a single data type gives a partial picture of the regulatory mechanisms, affecting the truthfulness of inferred results [25]. Data integration can overcome these limitations, allowing to build more accurate models, that are less prone to overfitting and more robust to noise and parameters perturbation [26].

To this aim, BNs provide an ideal probabilistic framework to handle heterogeneous data integration, and to incorporate biological functional information into the model as *prior knowledge*. Several structure learning methods have been tested to include prior knowledge in their search process, since the reconstruction of regulatory networks is computationally expensive [27, 28]. For instance, Imoto et al. [29] and Werhli et al. [30] represented biological priors in terms of energy function to evaluate the fitness of each learned network to the prior structure. Hartemink et al. [31], instead, included genomic location data as a model prior, forcing the search procedure to add arcs in a specific position, and discarding all graphs lacking these recommended edges. However, application of these algorithms is limited to small networks due to their complexity and high computational cost [32].

In this work, we present a data fusion approach for learning transcriptional Bayesian Networks in a high-dimensional space, exploiting heterogeneous *omics*-data integration, to determine the transcriptional architecture on a genome-wide scale. Our method implements a hybrid structure learning algorithm able to draw structural priors from Chromatin ImmunoPrecipitation followed by deep sequencing (ChIP-Seq) data. This type of epigenomic data produces a binding profile for each considered TF, consisting of all the target genes for which the TF is the transcriptional regulator. The integration of multiple genome-wide TF binding profiles allows reconstructing the circuitry of a regulatory network which captures the natural directionality of transcriptional flow.

Moreover, the algorithm exploits integrated gene expression data as evidence for both assigning prior probabilities to each individual transcriptional relation, and for learning the model parameters during its search process. This multi-layered -omics data integration can reveal topological hierarchies as a reflection of the transcriptional impact on gene regulation, which, to our knowledge, have not been investigated with a Bayesian learning strategy on a genomic scale.

We apply our novel framework to a chronic myeloid leukemia (CML) ChIP-Seq dataset, for gathering a data-driven prior knowledge to model the underlying transcriptional genomic interplay, whose overall structural consistency was further assessed through existing networks within the hematopoietic context. The performance of the proposed approach is then evaluated with other inference methods using as a benchmark a literature-derived transcriptional network of the yeast *Saccharomyces cerevisiae* that, for our purpose, is the only eukaryotic TRN available as gold standard with transcriptional cooperation level sufficiently complex if compared with ours.

## Results

We applied our data fusion approach to a Chronic Myeloid Leukemia (CML) dataset, using data-driven prior knowledge gathered from the integration of TFs ChIP-Seq binding profiles, in order to prove its ability to handle a real genome-wide transcriptional network. Given the noise linked to this experimental data source, we then tested the robustness of our hybrid learning algorithm to incorrect prior information, evaluating it on a gold standard regulatory network, from yeast *Saccharomyces cerevisiae,* and comparing its learning performance to other inference strategies, as described in the Methods section. Moreover, to further assess the structural consistency of the CML transcriptional model, we examined its regulatory patterns comparing them to other hematopoietic networks derived by another class of inference methods, which use DNA sequence information to predict TF regulations [6, 9].

### Performance assessment on high-throughput data

#### CML dataset

A collection of 65 TFs ChIP-Seq alignment data was retrieved from the Encyclopedia of DNA Elements (ENCODE) database [33] for the CML reference cell line K562. The binding profile of each TF was obtained through a bioinformatics pipeline, which included MACS2 peak calling [34], replicates consistency evaluation and peak-to-target assignment, in order to evaluate the statistical significance of the detected binding signals along the genome and to identify target genes. To further assess the consistency of the TF-gene interactions, each regulatory relationship was quantitatively weighted through a score-based method, which reflects the confidence of the considered binding event [35], discarding spurious interactions. The computational integration of all of the obtained genome-wide TFs profiles generated a genomic TRN composed of 20,876 nodes (65 TFs and 20,811 target genes), and 478,558 directed edges. Each edge was also weighted, using the score previously mentioned as a measure of the binding strength.

As first step, we dissected this genomic network into a TF-TF Component, characterized by 1827 edges between the 65 TFs, and a TF-Genes Component, which included the remaining network edges. Applying the BN design process (see Methods Section - Bayesian model definition) to the TF-TF Component, all weights (i.e. binding scores) associated with the arcs were sorted in decreasing order and evaluated within an iterative process aimed at finding a minimal connected directed acyclic graph (DAG). We obtained a whitelist of 1763 transcriptional relations and a DAG defined by 65 nodes and 64 interactions. This DAG was then combined with the TF-Genes Component to obtain a genomic transcriptional BN (TBN).

As a second omics data source, a compendium of microarray data from 122 CML patients was generated through the integration of five GE datasets, retrieved from GEO and ArrayExpress databases (GEO accessions GSE13159 [36], GSE47927 [37], GSE24739 [38]) (ArrayExpress accessions E-MTAB-2581 [39], E-MEXP-480 [40]). These data were normalized with the Robust Multi-array Average (RMA) technique [41], retaining the expression of those genes expressed in all the considered experiments. The TBN and the related whitelist were then integrated with this genomic expression panel and only relations among nodes for which the expression information was available were retrieved. The purpose was achieving a fully observable network, whose underlying distribution will be modeled as a joint multivariate Gaussian, where the conditional density of each variable given its regulators can be represented as a linear Gaussian model (see Methods Section).

**Fig. 1:**
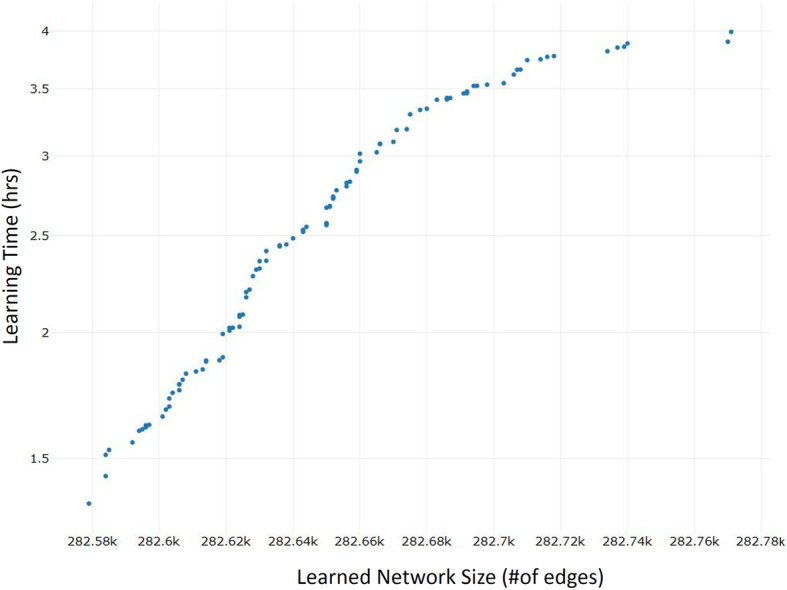
Performance evaluation of the hybrid structure learning algorithm on CML network. The execution time of the search strategy to learn the BN model structures is plotted against the number of edges of each learned genomic network

The resulting BN consisted of 11,986 nodes (of which 60 TFs) and 282,533 edges represented the initial structural input of our hybrid learning algorithm, together with the TF-TF arcs whitelist (1587 edges) for which the Pearson correlation between each TFs pair was estimated. After 100 runs of the algorithm, we collected 100 transcriptional BN models; the computational time required for learning all the obtained genomic networks on a single multi-core machine is shown in Fig. 1.

In order to obtain a transcriptional consensus BN and find consistencies across the learned structures, we estimated the robustness of each TF-TF edge from all the learned networks as a weight using Eq. (8), in order to rank these transcriptional relations, following the approach described in Methods Section - Consensus Transcriptional BN definition. We chose as a strict confidence threshold the weight value corresponding to the 5*th* percentile of the arcs weights distribution, to avoid the inclusion of edges with low confidence. The resulting consensus network was defined by 70 TF-TF edges; of these, 6 were present in the initial DAG but their directionality in the final TBN was reversed by the algorithm, as an effect of TRN regulatory loops.

**Fig. 2:**
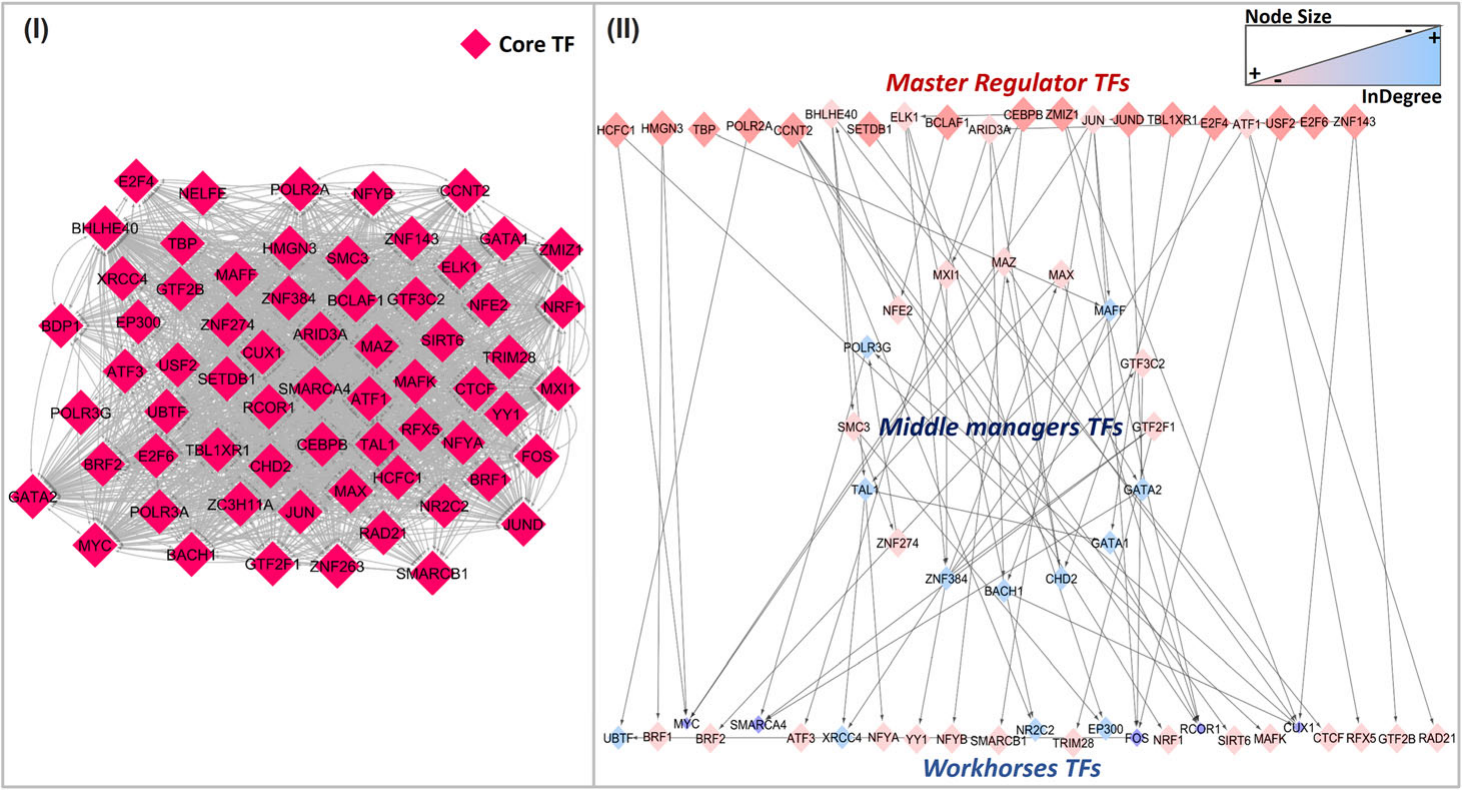
Comparison of transcriptional interactions models. Panel (I). Transcriptional regulations characterizing the TF-TF Component derived from ChIP-Seq data integration. Panel (II). Transcriptional hierarchy underlying the Consensus Bayesian Network. The color intensity and the size of TFs nodes are proportional to the incoming connectivity (e.g. small size combined with a darker color for a high number of incoming edges)

The connectivity of each consensus node was then analyzed computing topological statistics as out-degree and in-degree that evaluate the number of incoming and outcoming edges for a node, respectively. In particular, these measures were used to calculate for each TF the hierarchy height metric [6], to topologically mine the chain of command underlying the transcriptional flow of the network. We identified a three-layered hierarchy, as illustrated in Fig. 2 (II), representing the regulator activity of different TF classes, composed of 20 master regulator TFs, at the top, 16 brokers or middle managers, and the remaining 24 workhorses TFs, at the bottom.

This hierarchical organization was not detectable in the initial TF-TF component, derived from the sole integration of ChIP-Seq binding profiles, and due to the intrinsic complexity and compactness, the network has a typical “hairball” representation, as shown in Fig. 2 (I). As demonstrated by its high average node connectivity [42] ($\overline{k}\left( TF\_{TF}_{Component}\right)$ =20), all nodes are consistently interconnected to each other without a specific topological order. Clearly, the resulting regulatory schema emerged thanks to the transcriptomics data integration within the structure learning framework, where gene expression guides the learning phase in two steps of the algorithm search process. First, the sampling process is led by the arc extraction probability equivalent to the Pearson correlation calculated between each TFs pair, ensuring that edges tied to high correlated TFs have a greater probability to be included in the structural model, as reported in Supplementary Figure 2 in Additional File 1. Second, during the learning of the model structure, gene expression values were used for the estimation of parameters which define the probability distribution of each node given its parents regulators, as explained in Methods Section.

### Robustness evaluation to false prior information

#### Benchmark datasets

We retrieved all available transcriptional regulations in yeast among known TFs and target genes, which map to verified Open Reading Frames (ORFs), from the YEASTRACT [43] and Saccharomyces Genome Database (SGD) [44] repositories.

We used the normalized data from Spellman et al [45] as gene expression (GE) information, considering only those genes identified by the authors as cell-cycle regulated, and with a missing values rate less than 10%. We performed a *k*-nearest-neighbor imputation, obtaining a final complete dataset of 473 cell-cycle related genes expressed in 77 samples.

Combing the validated transcriptional binding information with GE data, we defined as ground truth a yeast regulatory network (yTRN) composed of 33 TFs and 437 target genes, and 3299 transcriptional regulations, 249 of which were TF-TF interactions.

To test the robustness of our method to incorrect prior information, we randomly added an increasing number of false edges to the yTRN, from 10 to 60% of the total number of TF-TF regulations. We considered each known interaction as true positive (TP), and every additional incorrect arc as false positive (FP). The performance of our method compared to the one of BANJO (Bayesian network interference with Java objects) [46] and ARACNe-AP [47] was evaluated for each FPs percentage, considering both the number of false edges included in the final model, and the fraction of true interactions among all inferred ones (precision). BANJO is a structure learning algorithm which, combining simulating annealing and a greedy search, finds and scores candidate networks inferring them from discretized expression data. ARACNe-AP uses instead the mutual information metric estimated from gene expression data input and data processing inequality to infer relations from a predefined list of TFs to their targets (see Competing Methods).

**Fig. 3:**
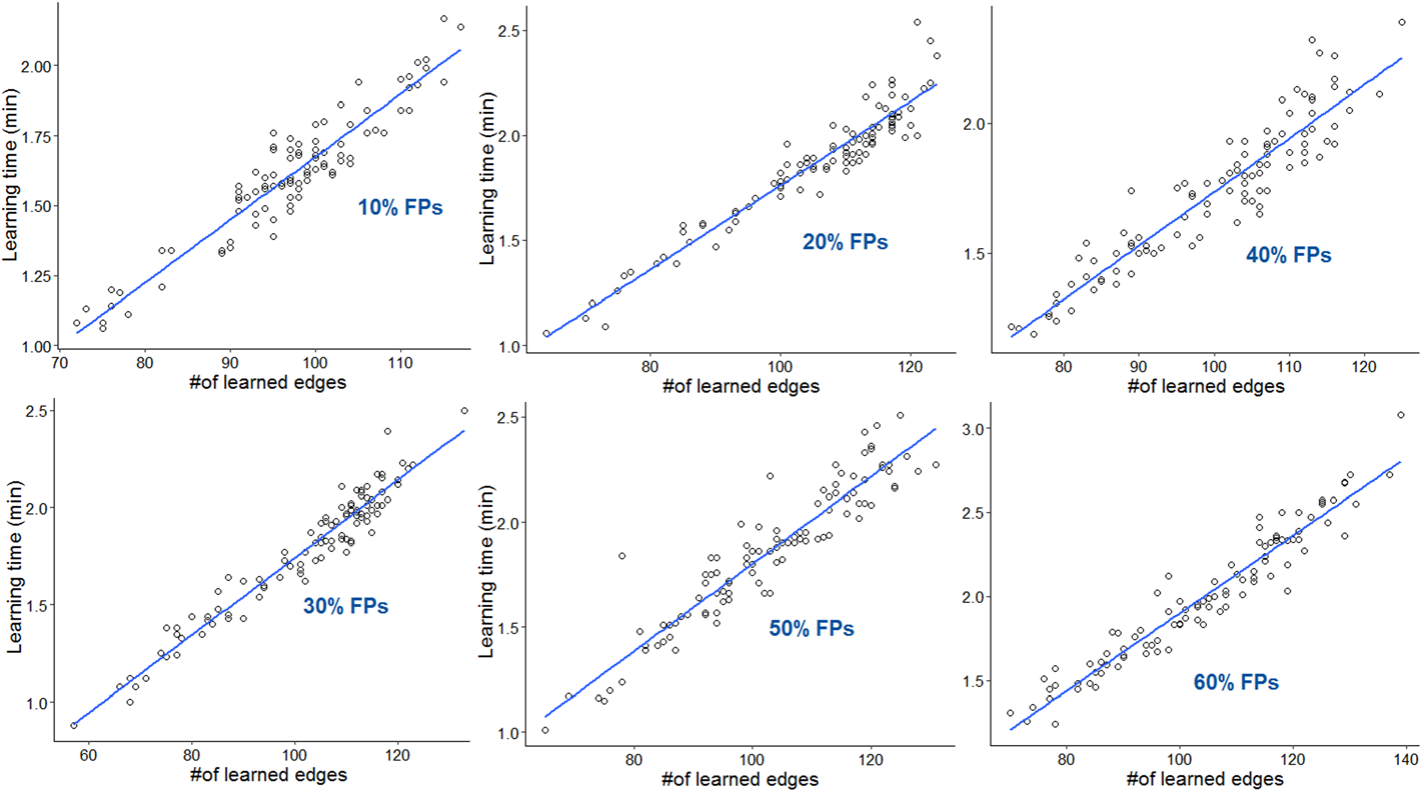
Performance evaluation of the hybrid structure learning algorithm on yeast network. For all the considered FP levels, we analyzed the execution time of the search strategy to learn the BN model structures, comparing it to the number of edges of each learned network

The yTRN underwent the BN definition procedure and was decomposed into a TF-TF Component of 33 TFs and 249 interactions, and a TF-Genes Component with 470 nodes and 3050 interactions. Using the option for unweighted transcriptional data, the TF-TF component was submitted to the iterative process, and a DAG with 33 nodes and 32 TF-TF interactions was then obtained. Combining it with the other Component, we defined the structure of the initialized model, consisting of 470 nodes and 3082 edges. This starting TRN and the arcs whitelist, whose dimension varied according to the considered FP rate, were used to test the proposed approach in all of the six incorrect prior conditions. We collected 100 learned transcriptional BNs for each tested FP percentage, and we evaluated the computational performance of our learning method on them, considering the time used by our algorithm to learn all the obtained networks, as illustrated in Fig. 3. The average computational time estimated on the total number of transcriptional BN models for all FP levels varied from 1.61 min to 2.00 min.

We then applied the “consensus” approach, described in Methods Section - Consensus Transcriptional BN definition, on each set of learned networks. For all the analyzed FP percentages, we selected the 25*th* percentile of the arcs weights distribution to find the confidence threshold for including only high confidence arcs in the related yeast Consensus BNs. The performance of our algorithm throughout all tested FP levels is reported in Table 1A and in Supplementary Figure 1.

**Table 1:** Summary of all results obtained from the comparison of described methods: (A) Data Fusion approach, (B) BANJO and (C) ARACNe-AP

Tested FP rate	Consensus BN size (#of edges)	% FPs added	Precision
A. Data Fusion Performance Results			
10%	50	8%	0.92
20%	58	12%	0.88
30%	56	10%	0.88
40%	60	12%	0.88
50%	69	11%	0.88
60%	76	12%	0.88
B. BANJO Performance Results			
10%	69	60%	0.41
20%	69	60%	0.41
30%	69	60%	0.41
40%	69	40%	0.59
50%	69	40%	0.59
60%	69	60%	0.41
**C.** ARACNe-AP Performance Results			
	1003	70%	0.3

BANJO was evaluated in each incorrect prior scenario taking as input data the discretized GE yeast dataset, the same initial DAG structures exploited by our approach on the yeast dataset, and a blacklist, to avoid gene-gene interactions and unrealistic regulations from genes to TFs. We ran BANJO using default parameters, and a fixed search time (5 h) as a stop criterion. All results are summarized in Table 1B.

ARACNe-AP cannot be evaluated under these incorrect prior conditions since it infers the network structure using GE data and a list of regulators (the considered 33 yeast TFs). Its Consensus network was obtained after 100 bootstraps from gene expression samples, using a MI threshold of 0.2989 estimated on the provided GE data. The number of FP edges calculated on the total consensus arcs is shown in Table 1C.

Comparing the Data Fusion (DF) approach with BANJO, which, as our method, exploits prior knowledge for guiding the learning phase, DF showed a higher precision and robustness despite the progressively higher FP rate included in each prior. Moreover, it does not require a blacklist, as instead for BANJO, to avoid the inclusion of unreliable regulations (i.e. relations from gene to TF), that, for this benchmark interactome of moderated size is composed of 205,391 edges. Clearly, for a genomic network with thousands of nodes, the blacklist definition composed of all relations from genes to TFs and from gene to gene would become more computationally onerous. DF outperformed also ARACNe-AP, which reached the lowest precision, highlighting how the expression alone makes difficult to discriminate between a highly correlated regulator-target gene pair and a true causal relationship. This learning method indeed relies on a single data source, and the only “prior” allowed is the list of regulators among which DPI procedure is applied to infer dependencies.

### Comparison with other hematopoietic transcriptional networks

To further assess the effectiveness of our framework and the overall quality of our transcriptional data-driven prior used for determining the final TBN structure, we next examined the extent to which ChIP-derived transcriptional patterns agreed on existing networks obtained with a different inference methodology class. Considering the hematopoietic context, we compared our learned CML interactome with other networks derived from K562 cell line data through inference methods that exploit genomic locations information to derive the network backbone [6, 9] but without a structure learning schema. In this analysis, structural comparisons were performed evaluating all the transcriptional relationships shared by common regulators among the considered networks.

#### DNaseI-footprinted hematopoietic transcriptional networks

**Fig. 4:**
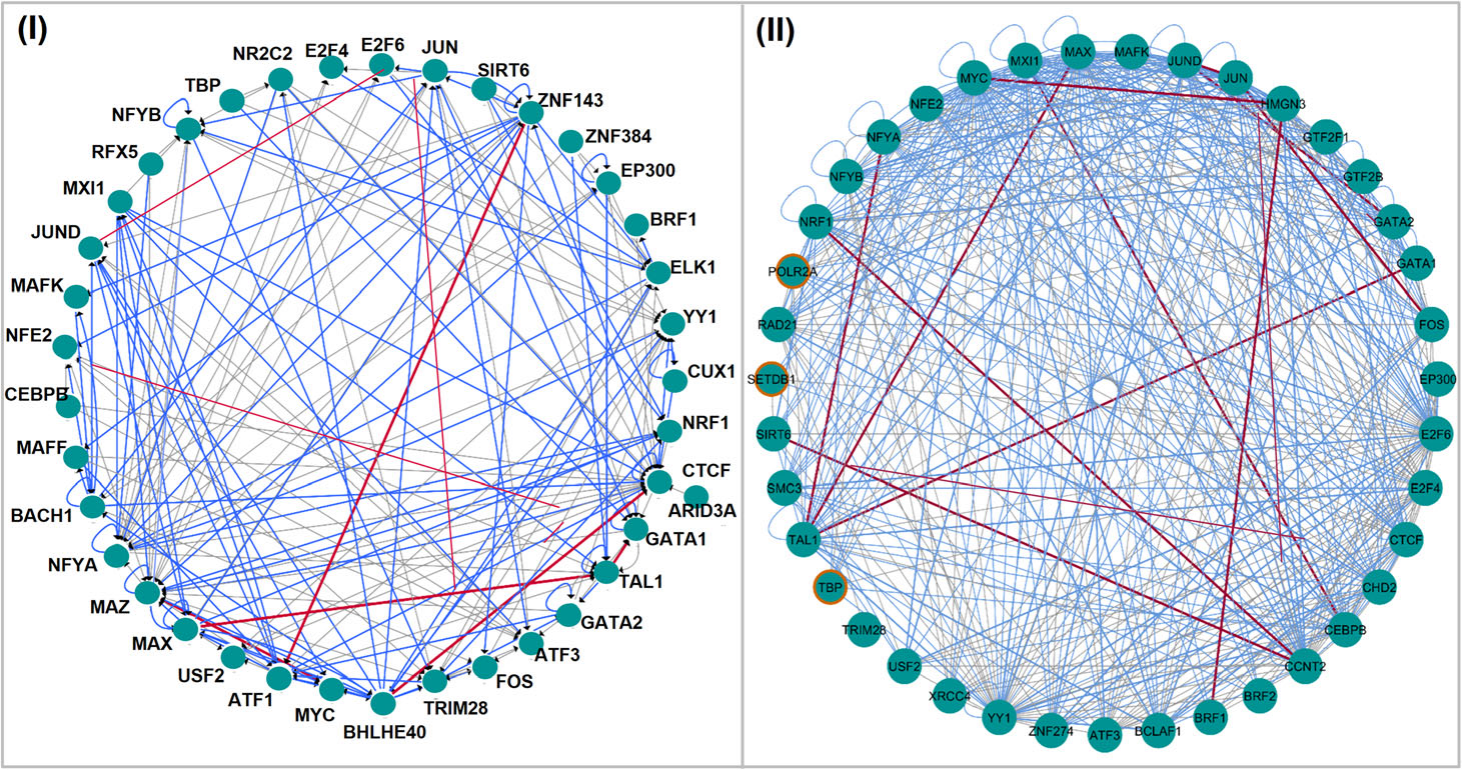
Structural comparison of K562 data derived transcriptional networks. (I). DNaseI-footprinted subnetwork. (II). TFs co association-based subnetwork. Common transcriptional dependencies are highlighted with blue edges and with red edges if the relationship is also included in the final TBN model. Orange bordered nodes are TFs considered only as targets in the TFs co-association network and not as source regulators like in the TBN

We exploited the transcriptional network obtained from DNaseI footprints of K562 data integrated with a predictive motif-based search of known TFs binding sites [9]. We extracted from this interactome all relations driven by the regulators in common with our TFs set, constituting a subnetwork of 38 TFs nodes and 165 edges, as depicted in Fig. 4, panel I. Considering all transcriptional dependencies shared by our ChIP-Seq derived regulations and the resulting DNaseI subnetwork, we reached a structural regulatory homology of about 60%, represented with blue edges in Fig. 4 (I), despite the difference of the applied inference techniques. In this set of common arcs, transcriptional relations of the final TBN are also included (red colored edges in Fig. 4 (I)). They represent high confidence arcs as a result of the selection process to which they underwent within our framework. The choice of transcriptional relationships is indeed determined by a trade-off between edge prior probabilities and the inherent ability to explain expression of a target gene in the learning phase of the hybrid algorithm and within the procedure for the consensus model outlining, by a quantitative score as a strength measure of dependencies across all learned models.

**Fig. 5:**
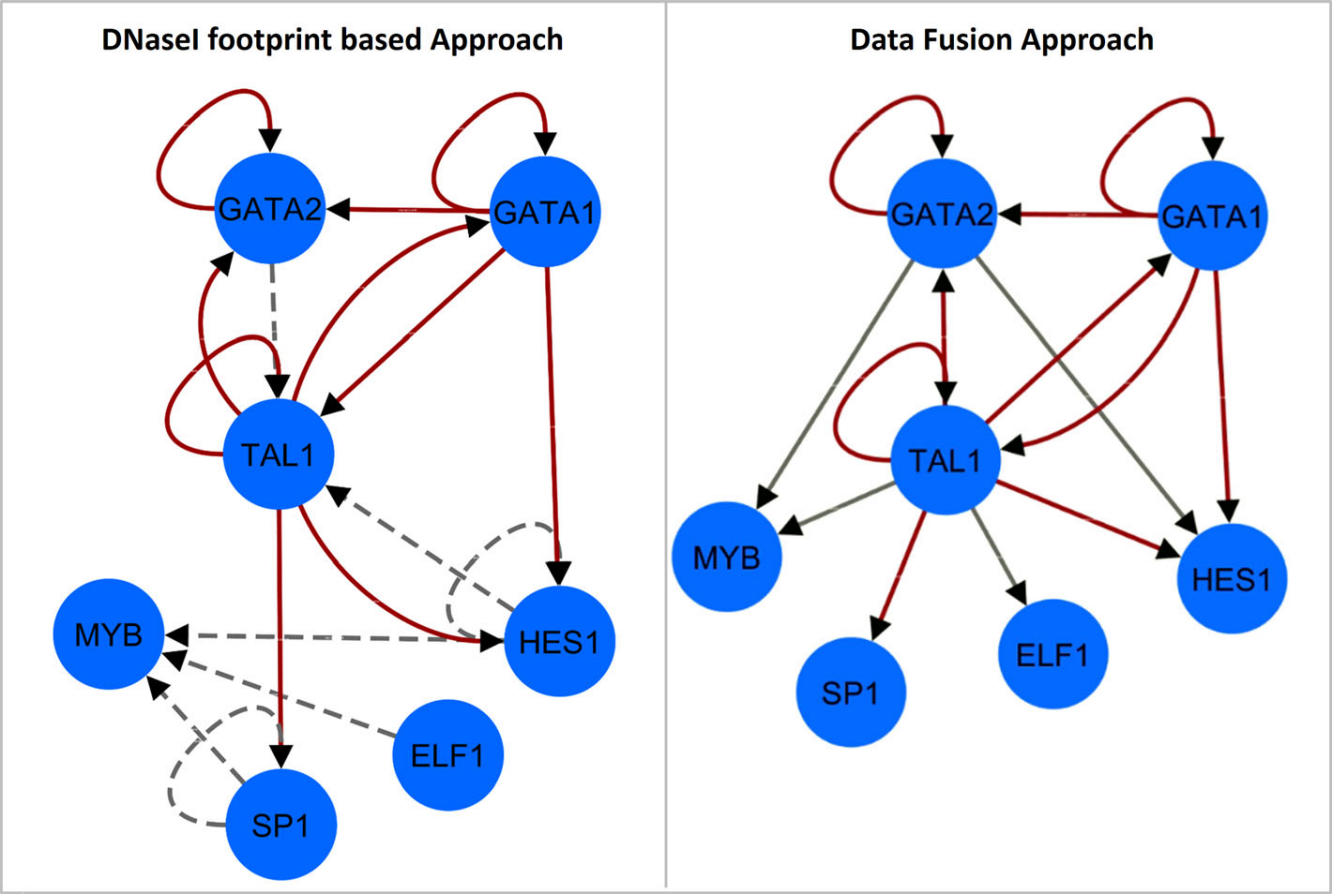
Major hematopoietic TFs network comparison. Shown are regulatory networks comprising edges among seven hematopoietic regulators derived by DNaseI footprints (left panel) and our proposed integrative framework (right panel). Transcriptional relationships are red colored to indicate common regulations or grey colored if they are specific for the considered network. Dashed grey arcs represent dependencies for which binding data are not available

Moreover, to further demonstrate the consistency of the transcriptional information enclosed in our prior, the comparative analysis was extended considering cross-regulatory interactions among the major transcriptional factors TAL1/SCL, SP1/PU.1, ELF1, HES1, MYB, GATA1 and GATA2, which have been extensively characterized for their lineage commitment role on hematopoietic cells [48, 49]. Examining the related networks obtained from the DNaseI footprinted interactome and our data, we obtained a structure similarity of 59%, as shown in Fig. 5. For SP1/PU.1, ELF1, HES1 and MYB, we cannot infer regulations (dashed grey arcs in Fig. 5), since we do not have the related ChIP-Seq binding profiles. On the other hand, some transcriptional relationships reconstructed only from our data (solid grey arcs in Fig. 5) are verified in literature within the hematopoietic context. For example, GATA2 play an essential role for the maintenance and proliferation of hematopoietic progenitor cells through tightly regulated interactions with other hematopoietic-associated TFs, including HES1 [50, 51], TAL1/SCL and MYB [52–54], patterns that, if altered, commonly lead to leukemogenesis. Another key factor is TAL1/SCL whose regulatory circuit, in which ELF1 and MYB take part, directs the expression of genes involved in the differentiation of blood cells types [48, 55–57]. These transcriptional dependencies are instead absent in the DNaseI derived network, highlighting the limitation of a knowledge-based transcriptional network reconstruction that, in this case, is constrained by the availability of known TF recognition sequences.

Given these considerations, we have also evaluated the subnetwork composed of all transcriptional interactions involving TAL1/SCL, GATA1 and GATA2, for which the binding profiles can be mined from both our data and the DNaseI derived network. Examining common regulations from shared TFs, we gained a 63% of structural concordance as represented in Supplementary Figure 3 in Additional File 1, and a 75% of edges as further source of transcriptional information included in our prior but missing in the DNaseI inferred regulations. Details about this analysis are reported in Additional file 1 (Major hematopoietic regulators subnetwork comparison).

#### TF co-association based hematopoietic transcriptional network

In order to perform this comparison, we considered the transcriptional network reconstructed from a TF co-association model learned from K562 ChIP-Seq co-binding matrix with a discriminative machine learning approach [6]. Evaluating common regulators between this network and our TFs group, we defined a core of 39 TFs and 464 edges, as depicted in Fig. 4, panel II, in which almost the entire interactions (~ 93%) are shared between the considered transcriptional models. To further highlight this structural overlap, we have also investigated the underlying regulatory hierarchy using both the hierarchical score, applied on the TFs co-association derived network, and the hierarchy metric, used on our model. We obtained again a high degree of similarity in terms of level assignment, confirming our structural regulatory diagram. Results of this analysis are described in Additional file 1 (TF co-association based hematopoietic transcriptional network: Hierarchical comparison).

Among common interactions, consensus relationships verified in the previous analyses were also supported by this comparison. TAL1-CEBPB-GATA2 are primary-interacting partners of GATA1 which guide lineage-specific differentiation of hematopoietic cells. GATA1 has been shown to recruit TAL1 at several erythroid enhancers [58], regulating gene expression after being directed to a distinct subset of genomic binding sites in multi-lineage cells via its association with different complexes containing master regulators such as GATA2, CEBPB and RUNX1 [59, 60]. Moreover, GATA1 has a competitive behavior for the binding in regulatory complexes with GATA2, with which it is frequently co-associated [61]. Together with MAX, these factors constituted a regulatory circuit involved in erythrocyte and myeloid differentiation [62]. In addition to TAL1-CEBPB, another novel pairing CCNT2-HMGN3, identified and validated in the Gerstein et al. study [6], is also present in our final model. The Activator protein-1 (AP-1) complex consists of JUND-JUN-FOS factors, all of which are known to form heterodimeric protein aggregates with each other, which promote myeloid differentiation, and genetic lesions affecting their expression have been associated to the leukemogenesis process [63, 64].

Furthermore, in the TF co association-based network, three of the 39 core TFs were considered as targets (POLR2A, TBP and SETDB1, orange bordered nodes in Fig. 4, II) and not as source TFs which can regulate other nodes like in our network, missing a part of regulatory information. POLR2A and TBP are key component of the core transcriptional machinery whose interaction with other hematopoietic co-regulators such as CEBPB, SP1, RUNX-related factors can modulate gene expression programs during myelopoiesis [65]. SETDB1 is instead a gatekeeper of tumor survival whose chromatin remodeler role is recently emerged as a potential therapeutic target for immunotherapy to avoid leukemic cells evasion from immune system [66, 67].

## Discussion

In the era of ‘Omics’, data integration represents a challenging tool to deliver more comprehensive insights into the biological system under study, helping to translate novel molecular knowledge into improved disease understanding. In particular, going deeper into cancer deregulated gene expression programs, investigating their first level of regulation, where the transcriptional determinants act on a genome-wide scale, may help to define the molecular signatures driving the patient’s phenotype. To this aim, the development of a robust computational approach able to deal with omics data heterogeneity and with this biological complexity is mandatory.

In this work, we proposed a data fusion approach which exploits multi-layered genomic data integration, allowing to model large-scale transcriptional networks within a Bayesian formalism. Our hybrid structure learning strategy allows to use ChIP-Seq transcriptional binding profiles as prior information, to both initialize the model structure and to constrain the search space. In particular, it models the natural directionality of the transcriptional flow, also evaluating edges which belong to feedback regulatory loops, and whose direction may be reversed by the algorithm. The learning procedure also exploits gene expression (GE) data integration, which acts on the initial search phase, (*i*) with the correlation, as a sampling probability tied to the arcs whitelist, allowing that a higher correlation will be translated into a greater prior probability for a transcriptional dependency to be included in the final model; and then (*ii*) on the estimation of the model parameters, specifying how combination of TFs functionally regulates the expression of their targets. We proposed a prior-based approach that it first reconstructs the regulatory skeleton and then refines the network structure using condition-specific expression data, prioritizing the underlying regulators. Using this joint learning schema, we obtain increased accuracy of the reconstructed transcriptional networks compared to those approaches which rely only on a single data source (i.e. GE data), such as ARACNe-AP. Despite these inference methods are widely used, as already pointed out [68], the expression alone makes it difficult to gain mechanistic insight between a highly correlated regulator-gene pair and a true causal relationship between them. In this framework, learnt models that are generated from each learning run of the algorithm underwent to a “Consensus” definition procedure, which ensures that only consistent dependencies appear in the final transcriptional model, reducing the occurrence of weak relationships. In addition, the estimated correlation is converted in probability linked to each edge, as a further measure of ‘robustness’ of the considered binding event.

We applied our data fusion method to a CML -omics dataset, to test its computational ability to learn a genomic transcriptional network and model its complex transcriptional interplay. Although the mutational causative event of the considered disease is known to be the BCR-ABL1 gene fusion, the underlying transcriptional architecture has not been deeply investigated yet. Therefore, we wanted to mine the molecular mechanisms linked to the considered disease maintaining a genome-wide overview. To this end, we used integrated ChIP-Seq binding profiles from K562 cell line that is a representative in-vitro model of the CML, combining the resulting functional readouts with gene expression data from untreated CML patients, allowing to emerge only disease linked processes. The proposed learning strategy enabled us to identify a stratified hierarchy in the final consensus transcriptional Bayesian Network, representing the overall system-level regulatory wiring, which was undetectable in the initial CML transcriptional network. Indeed, the starting TRN, obtained from the integration of a single data type, the ChIP-Seq binding profiles, showed a high compactness and a complex connectivity, emerging from TF-TF interactions, due to the cooperative behavior of TFs, difficult to translate into a meaningful biological inference. The three-layered hierarchy instead can be interpreted as the effect of regulator impact of different TF classes (master regulators, middle managers and workhorses) on gene expression cellular programs, since the learning phase of the hybrid algorithm is driven by the transcriptome expression. These specified TF levels collectively control the non-regulator gene targets, lying in a lowest fourth layer that, due to its large size, cannot be graphically showed.

The correlation between the topological and functional aspects of TF, established within this hierarchy in a genome-wide perspective, represents an interesting novel result for the considered disease that could be further experimentally investigated. A pivotal role of the epigenetic regulation is also emerged from these transcriptional interactions, whose importance and implications for leukemia have been recently emphasized [69]. Moreover, most of these transcriptional dependencies were confirmed in other hematopoietic transcriptional networks differently inferred from K562 data sources. For example, the SETDB1 TF that in this context has been topologically classified as a master regulator (MR), is characterize also by an epigenetic activity, regulating gene expression via chromatin remodeling. Aberrant SETDB1 functionality and the related altered epigenetic changes have been shown to promote silencing of tumor suppressor genes, and thus contributes to enhance tumor growth and metastasis [70]. Moreover, SETDB1 maintains hematopoietic stem cells, restricting the activation of non-hematopoietic genes in normal conditions [71] while, when deregulated, it enables leukemia cells to evade innate immune controls allowing them to expand [66]. Our result highlights the importance of this TF, confirmed by its emerging role as a promising therapeutic target for several types of cancer [72, 73], including other forms of acute and chronic leukemia.

Another MR, the CEBPB TF, within the hematopoietic system is effectively indicated in the literature with as playing the role of MR, expressed at high levels to regulate genes involved in immune and inflammatory responses. Under stress conditions, such as cancer microenvironments, CEBPB is involved in BCR–ABL1 mediated myeloid expansion and leukemic stem cell exhaustion in the CML chronic-phase [74]. The MR ZNF143 was observed to bind CEBPB and other C/EBP factors, whose interactions are required for a balanced expression in myeloid cells and for granulocytic differentiation of myeloid progenitors [75]. Members of the Jun family (JUN and JUND), that are key subunits of the transcription factor AP-1, are designated as MRs in healthy and cancer cells [76], given their crucial role in cell cycle progression, differentiation and programmed cell death. Not surprisingly, they are frequently overexpressed in leukemia, and their leukemogenesis actions are BCR-ABL1-induced [77]. Despite RAD21 and SMC3 TFs belonging to the same cohesin complex involved in DNA damage repair and whose composing genes are frequently mutated in myeloid neoplasms [78], these regulators are located at different network layers as a result of their different effects on their regulating modules. The same observation can be drawn for the heterodimeric complex composed of MAX and MYC genes, situated in the central and lower part of the hierarchy, representing their sequential recruitment necessary for regulating hematopoietic homeostasis [79]. Furthermore, MYC maps in the same layer of YY1, another known cooperating partner, whose expression alteration impacts on the MYC oncogene function. CTCF also co-localizes with RAD21 and together with SMC3 are commonly associated with insulator elements to mediate long-range interactions affecting the higher-order chromatin structure [80]. SMARCB1 and SMARCA4 interacting TFs lie in the same hierarchical level; both belong to the SWI/SNF complex as chromatin remodelers, playing an important function in pluripotency and cellular reprogramming. Recently, their involvement in maintaining oncogenic gene expression program in myeloid leukemia, in particular for the tumor suppressor SMARCB1, have been demonstrated [81]. GATA1 and GATA2 are two fundamental TFs which play a crucial role in gene regulation during development and differentiation of hematopoietic cells. They belong to the same hierarchical layer, reflecting their sequential molecular recruitment; it is indeed know that GATA2 binds the promoter region of GATA1 whose expression can be repressed in the hematopoietic stem and progenitor cells [53]. ATF1 is a master regulator, as detected in our model, often traslocated or overexpressed in blood malignancies, promoting leukemic cells expansion and resistance [82]. TBL1XR1 is a factor required for the activation of multiple intracellular signaling pathways important for hematopoietic cells fate, not surprisingly identified in this context as master regulator. A variety of genomic alterations was identified on this gene in several forms of leukemia, and its loss observed in recent studies has been proposed as a potential therapeutic target [83]. E2F4 and E2F6 play an essential function in specifying lymphoid subtype, orchestrating the activation of essential cell cycle progression genes and other key TFs, such as EBF1, required for normal and malignant B-lymphocyte development [84, 85]. Moreover, these E2F proteins have been found to co-associate with HCFC1/HCF-1, another TF that, in our hierarchy, was classified as master regulator, inducing histone methylation and transcriptional activation and contributing to leukemogenesis [86]. Recently, it has been shown that expression of the BCLAF1 MR is increased in leukemia blasts relative to normal precursor populations and suppression of this TF highlighted its potential in neoplastic self-renewal program, causing reduced proliferation and leading to induction of differentiation to a dendritic cell fate [87, 88]. HMGN3 belongs to a family of chromatin remodeling proteins that are enriched in aggressive cancers and stem cells, due to their role in maintaining nuclear organization critical for stem cell properties, both during development and oncogenesis. These factors are frequently overexpressed in leukemia, enhancing aberrant gene transcription [89, 90]. Underlying this proposed regulatory schema clearly emerged a key role of the epigenetic regulation, whose involvement in leukemia-related processes has become of clinical relevance in the last years.

We then benchmarked our procedure against the yeast transcriptional network, demonstrating the robustness of the method to an increasing amount of false positive prior information that can also interpreted as a noisy source, intrinsic characteristic of experimental data. The validation of our procedure was performed only on the yeast dataset, because only a few experimentally verified eukaryotic transcriptional networks are commonly available as gold standards, like yeast *S. cerevisiae* and *E. coli*. This last one has a transcriptional network not sufficiently large and complex to apply our hybrid learning strategy, since the TF-TF counterpart with 214 TF-TF interactions annotated with strong evidence in RegulonDB database [91] is poorly enriched of TFs coregulations. The proposed data fusion technique is tailored for investigating complex transcriptional networks enriched of many coregulatory interactions, as human transcriptional networks, since these co-binding events are initially exploited by the algorithm to define its structural priors and the Bayesian model, and during the learning phase to add or reverse an existent arc. This implicates that the final probabilistic model will include only high confidence and consistent relations across all learned structures, as a selection of all initial coregulations.

Given the peculiarity of the proposed structure learning strategy, it is difficult to find similar published approaches for the validation process. Despite some common features, we excluded a recent Bayesian structure learning tool, bnlearn, since it forces arcs designed as structural prior (specified through a *whitelist*) to be included in the final model, preventing the addition of any other extra transcriptional regulation. For these reasons, we performed a comparison with the hybrid search algorithm implemented in BANJO, and with ARACNEe-AP, an ARACNe based approach for reconstructing transcriptional regulatory network. Our algorithm outperforms both methods, producing a significant improvement in structural accuracy, even with a progressively higher FP rate.

ARACNe-AP bases its structural reconstruction only on a single source of data (GE data), and this penalizes the correctness of the inferred transcriptional relations, 70% of which are FP predicted interactions. On the other hand, BANJO allows specifying a structural prior, but its implemented constraints and parameters, whose setting is not trivial (i.e. initialTemperature, coolingFactor, reannealingTemperature, etc.), does not enable to perform an accurate learning.

From a computational perspective, our approach is fast and scales well, thanks to its search method, particularly appropriate for parallel computation, and for the learning phase, based on local learning, while most Bayesian reconstruction methods, which use prior knowledge, are not practical for large networks. Our algorithm does not constrain the number of interacting variables or the maximum number of parents for each variable, as done in other methods [31, 92] and in BANJO, for which is advised to set this threshold less than seven, due to memory requirements needed for the learning. Moreover, it does not require a list of forbidden arcs (*blacklist*), like BANJO or bnlearn, whose definition for large-scale transcriptional networks is equivalent to 2^*n*^ (where *n* is the number of genes) interactions to exclude.

This data fusion method is designed to exploit data integration at different levels analyzing the resulting combined information in a unified framework, to gain insights into molecular signatures potentially driving disease phenotypes. In this study, we used high-throughput genomic datasets as the case of greatest complexity to present the ability of the approach in handling genome-wide transcriptional networks, whose modeling represents a novelty in the field of Bayesian structure learning algorithms.

The proposed methodology was conceived to use a genomic transcriptional interactome, classically enriched of co-regulations among TFs, as a primary integrative source for Bayesian model initialization and structural priors definition. Interactions among TFs, the main property of transcriptional networks, is the only feature required for the input transcriptomics data, making the learning algorithm highly adaptable to different transcriptional sources that infer TF binding events either with a direct approach (e.g. ChIP-Seq) or with an indirect one. Indeed, given the experimental heterogeneity and availability of published transcriptional data, the starting transcriptional network can be built in different ways, not only pooling together several ChIP-binding profiles, as was done in this study, but for example integrating ChIP data with TF-TF regulations derived from biological interactions databases such as STRING [93] or BIOGRID [94], or with motif-based search to find core regulators of the network, also combined with accessible chromatin profiles obtained from ATAC-Seq and DNase-Seq assays. The adaptability of our prior-based approach allows to integrate different –omics types of regulatory evidences to infer a genome-wide transcriptional network, whose structure, defined by TF dependencies, is then refined with condition-specific expression data, performing a comprehensive characterization of the potential factors driving disease transcriptional signatures.

## Conclusions

In this work, we proposed a data fusion-based approach that, exploiting -omics data integration, is able to reconstruct genome-wide transcriptional networks and, using Bayesian modeling, enables a probabilistic assessment of the underlying structure within the hybrid learning algorithm. An innovative aspect of our method is that the structural properties of the initial reconstructed network are defined from ChIP-Seq data and are used as prior knowledge. Combining such informative priors with a search and score schema both at the local and global levels of the structure model, the algorithm efficiently handles genome-scale networks. Moreover, the “consensus” approach allows including only high confidence learned interactions in the final transcriptional BN.

We studied the transcriptional landscape of chronic myeloid leukemia, which, to our knowledge, has not been investigated at a genome-wide level with a multi-layered Bayesian framework. The obtained findings demonstrate that our method uncovers interesting transcriptional interactions, relating the effective regulatory impact of TFs on gene expression with topological network properties.

The validation results showed the robustness of the proposed approach to noisy data, such as omics sources, and to prior knowledge with limited reliability, here given as increasing fractions of false transcriptional interactions in the priors.

The developed method is divided into well-defined steps in order to be applicable to other case studies, e.g. adapting the iterative procedure for finding the initial DAG to weighted or unweighted arcs while the TRN reconstruction phase, or the score threshold as ending criterion of the learning algorithm, varying the sampling edges set size, or the confidence threshold for the “consensus” approach.

This makes the developed data fusion approach an ideal framework for integrating potentially noisy complementary data, and a data-driven platform for transcriptional regulatory network inference.

## Methods

**Fig. 6:**
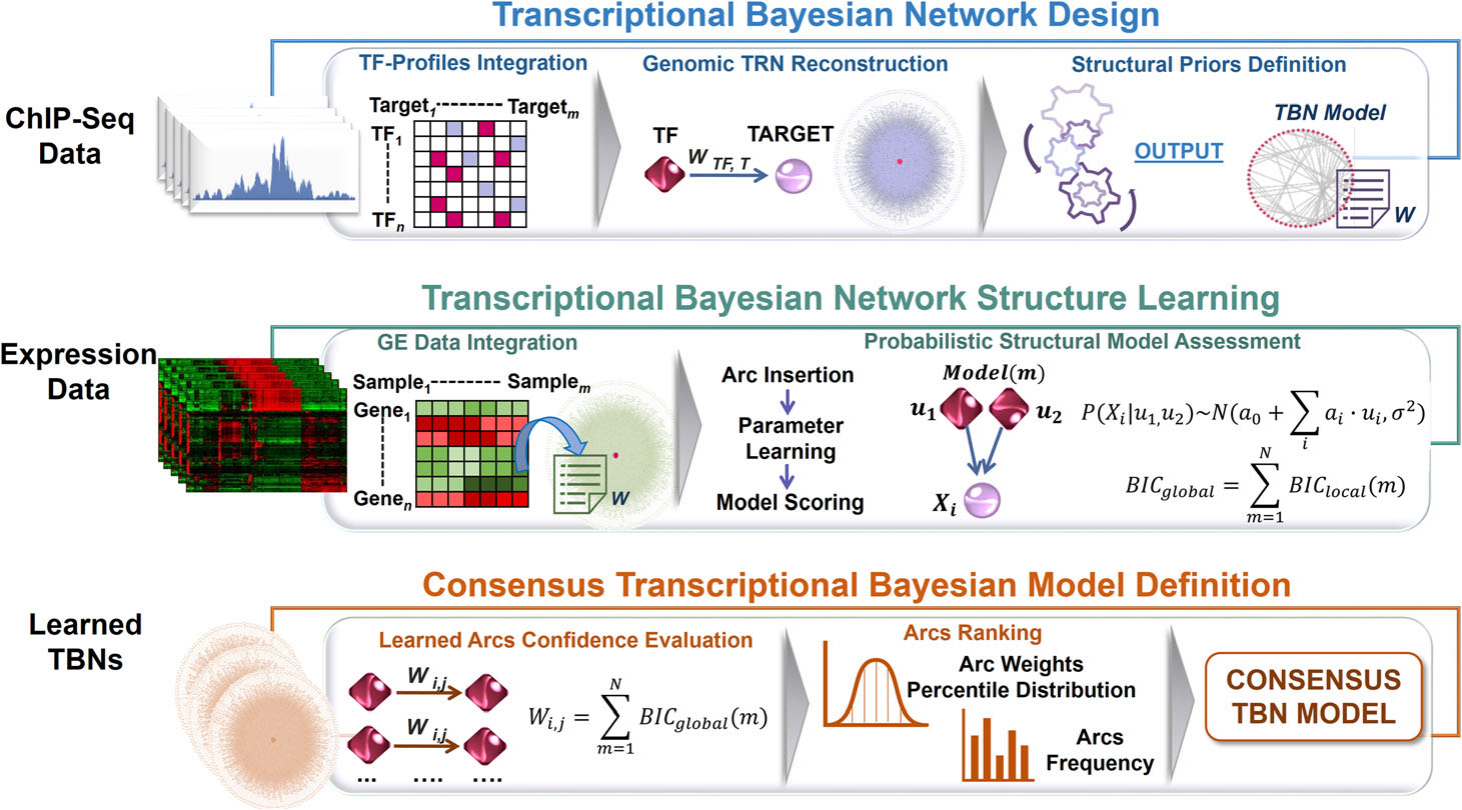
Overview of the Data Fusion approach. The procedure is structured on three main parts defined as (i) the reconstruction of the transcriptional network and its Bayesian modelling (upper panel), (ii) the model integration with transcriptomics data for its probabilistic assessment through the hybrid structure learning algorithm (central panel), and (iii) the consensus definition process aimed at identifying high confidence interactions among all learned transcriptional dependencies

In this section, we present our data fusion approach, whose main steps are depicted in Fig. 6. Its multi-step procedures rely on –omics data integration, exploiting data complementary to jointly investigate transcriptional regulations at genomic level under a unified Bayesian framework. The proposed method firstly uses ChIP-Seq data to reconstruct a genomic regulatory skeleton, from which the implemented hybrid algorithm draws structural priors and evidence from integrated expression data to probabilistically assess the transcriptional network structure. The final evaluation on transcriptional relations obtained from all the learned models ensures that only consistent dependencies will characterize the final transcriptional Bayesian model.

The following sections provide a description of the aforementioned Bayesian framework in which our developed hybrid algorithm lies, of its structural constraints definition and of the learning strategy.

### Bayesian modeling framework

A Bayesian network model is a graphical representation of the joint probability distribution of a set of random variables *X* = {*X*_1_, …, *X*_*n*_}. The encoding of this probability distribution is defined by a network structure S and a set of model parameters Θ, which describe the probability distribution of model’s variables [95]. Model structure S is represented as a directed acyclic graph (DAG), whose vertices (or nodes) are the random variables, and whose conditional dependencies are described by directed edges. In particular, each variable is assumed to be independent of its non-descendants given its set of parents, denoted as ***pa***(*X*_*n*_). Under this Markov assumption, the joint probability distribution of all nodes of the model is given as

1 $$ P(X)=\prod \limits_{i=1}^nP\left({X}_i\right|\boldsymbol{pa}\left({X}_i\right)=\prod \limits_{i=1}^n{\theta}_{X_{i\mid pa\left({X}_i\right)}} $$

where each variable *X*_*i*_ is described by a set of parameters (θ_i_) which defines the variable distribution conditional on its parents.

Within our transcriptional network context, a BN model represents the regulatory relationships among transcription factors (TFs) and from TFs to genes. An edge denotes an observed transcriptional regulation relationship between the considered nodes. All the variables of the model are real valued, and the joint distribution is assumed to be a multivariate Gaussian [96]. The conditional density of each variable *X*_*i*_ given its parents ***pa***(*X*_*i*_) = {*U*_1_, …, *U*_*k*_}, can be represented as a linear Gaussian model Eq. (2).

2 $$ P\left({X}_i|{u}_1,\dots, {u}_k\right)\sim N\left({a}_0+{\sum}_i{a}_i\bullet {u}_i,{\sigma}^2\right) $$

That is, *X*_*i*_ is normally distributed around a mean that depends linearly on the values of ***pa***(*X*_*i*_); the variance of this Normal distribution is independent of the parents’ values. In this representation ${\theta}_{X_i\mid \left\{{u}_1,..,{u}_k\right\}}=\left\rangle <,{a}_0,\dots, {a}_k,\sigma \right\rangle >$.

Given a dataset *D* = {*D*_1_, …, *D*_*n*_} where *D* is an instantiation of all the variables in *X*, learning BN structure from *D* corresponds to finding a model structure that best fits the observed data.

Finding the optimal BN represents an NP-hard (nondeterministic polynomial-time) problem that has been approached with constraints-based and score-based structure learning methods [97]. The former strategy exploits conditional independence tests to construct a partially oriented graph, retaining or rejecting candidate edges; the latter uses a scoring function to assign a network score reflecting its goodness of fit, which the algorithm then attempts to maximize. Both strategies scale to large networks poorly, because the number of possible graph structures or tests rises exponentially as the size of the network increases.

Hybrid algorithms, which are another class of structure learning methods, combine the characteristics of both aforementioned approaches to maximize their advantages. Typically, they start with a constraint-based search to find the skeleton of the network and then employ a score-based scheme to identify a high-scoring network structure. Our algorithm follows this search and score paradigm, optimizing the learning in order to manage genome-scale networks.

### The hybrid Bayesian network structure learning algorithm

The learning procedure is preceded by two fundamental phases of *omics-*data fusion in order to gather informative priors from genome-wide binding data and to define the parameters space from integrated gene expression profiles.

The starting point of this framework is a Transcriptional Regulatory Network (TRN) obtained from the integration of TF binding data. It is defined as a directed graph *TRN* = ⟨*V*, *E*⟩, where V is the set of TFs and genes vertices, and E is a set of ordered pairs of edges composed in turn by two subsets, describing the regulatory interactions between TFs (E_1_) and from TFs to genes (E_2_).

$$ TRN=\left\langle V,E\right\rangle where\kern0.5em {\displaystyle \begin{array}{c}V=\left\{{TF}_1,\dots, {TF}_i,{G}_1,\dots, {G}_k\right\}\\ {}E=\left\{\begin{array}{l}{E}_1=\left\{\left({TF}_1,{TF}_2\right),\dots, \left({TF}_i,{TF}_j\right)\right\}\;{\forall}_i{\forall}_j,i\ne j\\ {}\kern1.44em {E}_2=\left\{\left({TF}_1,{G}_1\right),\dots, \left({TF}_i,{G}_k\right)\right\}\end{array}\right\}\end{array}} $$

First, the TRN is converted into a Bayesian model, defining its structural constraints. The obtained transcriptional BN is then integrated with an expression data compendium, to achieve a fully observable network whose structure and parameters will be learned by our algorithm.

#### Bayesian model definition

The Bayesian learning procedure starts from an initial directed acyclic graph (DAG), which does not allow loops. Transcriptional networks are characterized by many loops of regulation, a typical characteristic of the dynamic crosstalk among TFs, through which they modulate both the expression and the activity of other TFs [98]. Since the TRN is enriched in these regulatory patterns, to match the acyclicity Bayesian constraint, the proposed approach exploits the property of TRNs, whose regulations can be divided in turn in two subsets, defining the interactions between TFs and between TFs and genes, respectively.

$$ {\displaystyle \begin{array}{l} TF-T{F}_{Component}\\ {}{TF}_C=<{V}_{T{F}_C},{E}_{T{F}_C}>\kern0.28em where\kern1.32em \left\{{E}_{T{F}_C}=\begin{array}{c}{V}_{T{F}_C}=\left\{{TF}_1,\dots, {TF}_i\right\}\\ {}\left\{\left({TF}_1,{TF}_2\right),\dots, \left({TF}_i,{TF}_j\right)\right\}\kern0.28em {\forall}_i{\forall}_j,i\ne j\end{array}\right\}\\ {}\\ {} TF- Gene{s}_{Component}\\ {}{TF}_{G_C}=<{V}_{T{F}_{G_C}},{E}_{T{F}_{G_C}}> where\kern1.8em \left\{\begin{array}{c}{V}_{T{F}_{G_C}}=\left\{{TF}_1,\dots, {TF}_i;{G}_1,\dots, {G}_k\right\}\\ {}{E}_{T{F}_{G_C}}=\left\{\left({TF}_1,{G}_1\right),\dots, \left({TF}_i,{G}_k\right)\right\}\end{array}\right\}\end{array}} $$

**Fig. 7:**
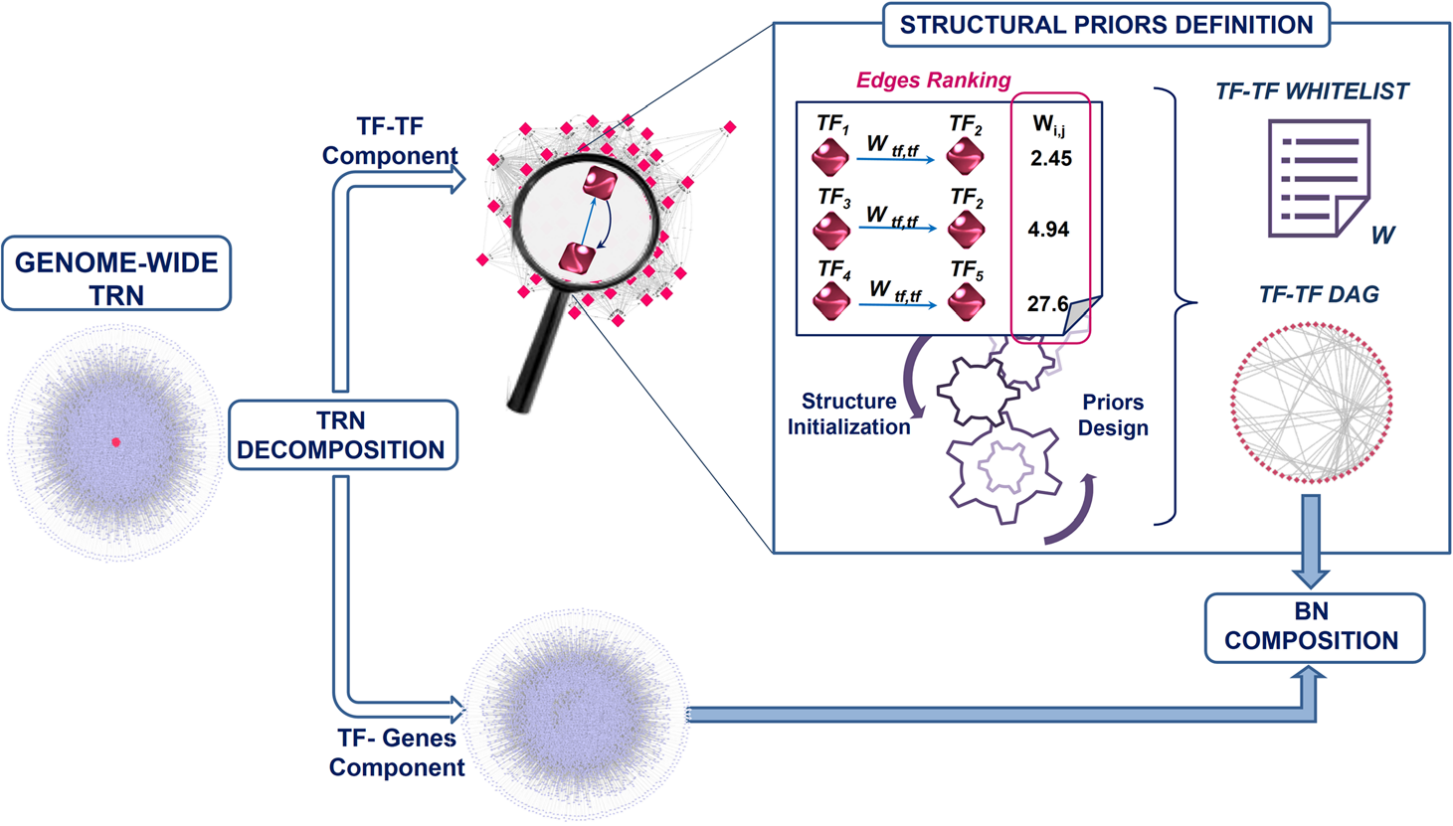
Transcriptional BN definition. Decomposition of a genome-wide Transcriptional Regulatory Network (TRN) allows to operate on the TF-TF Component, characterized by many regulatory loops, e.g. feedback loop (as shown in the magnifying glass) to initialize the BN structure model and its structural constraints. The obtained DAG is then combined with the TF-Genes Component to define a genome-scale transcriptional BN

As shown in Fig. 7, the TRN is decomposed into its fundamental parts, in order to be transformed into a BN: a TF-TF Component, consisting in TF-TF edges (which may contain regulatory loops), and a TF-Genes Component, consisting of edges from TFs to genes. The former then undergoes an iterative process aimed at initializing the model structure and defining the priors of the algorithm, while removing loops. Within this scheme, the procedure first evaluates the edges between TFs, ranking and sorting them in decreasing order if they are weighted, otherwise it shuffles all the arcs and assigns an equal weight to them. The process then removes one arc at a time, starting from edges with lower weight (if arcs are weighted), otherwise randomly extracting an arc, and checking, at every iteration, if the TF-TF Component is still full connected. The procedure ends when at least a minimal connected DAG is found. All the TF-TF edges excluded from this structure initialization constituted an *arcs whitelist* (W), which will represent the search space of the algorithm.

**Fig. 8:**
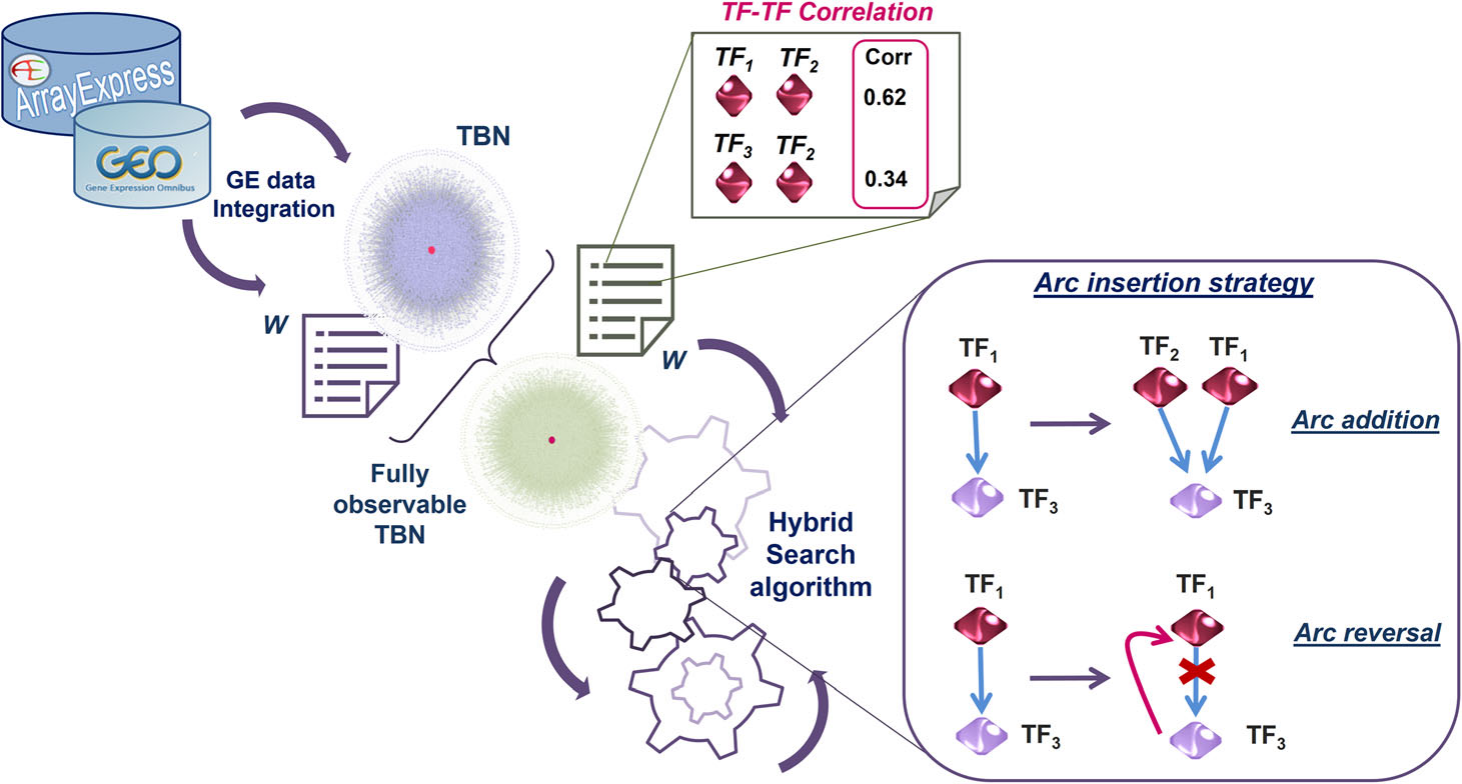
GE data integration of the transcriptional BN. GE data were integrated in the Transcriptional BN (TBN) and in the related arcs whitelist, defining them as inputs to the search algorithm. The box on the right highlights a peculiarity of the learning procedure, described in the Methods Section

The resulting DAG is joined with the TF-Genes Component, to obtain again a genomic transcriptional network, but designed as a Bayesian model (TBN). As a second step, the TBN is then integrated with gene expression (GE) data, as shown in Fig. 8 below, in order to obtain a fully observable BN. This transcriptomic data source is also used to calculate the correlation between TFs included in the whitelist, since this measure will be exploited as a sampling probability for each whitelisted arc extracted by the hybrid algorithm and evaluated in the TBN.

#### The structure learning strategy

**Fig. 9:**
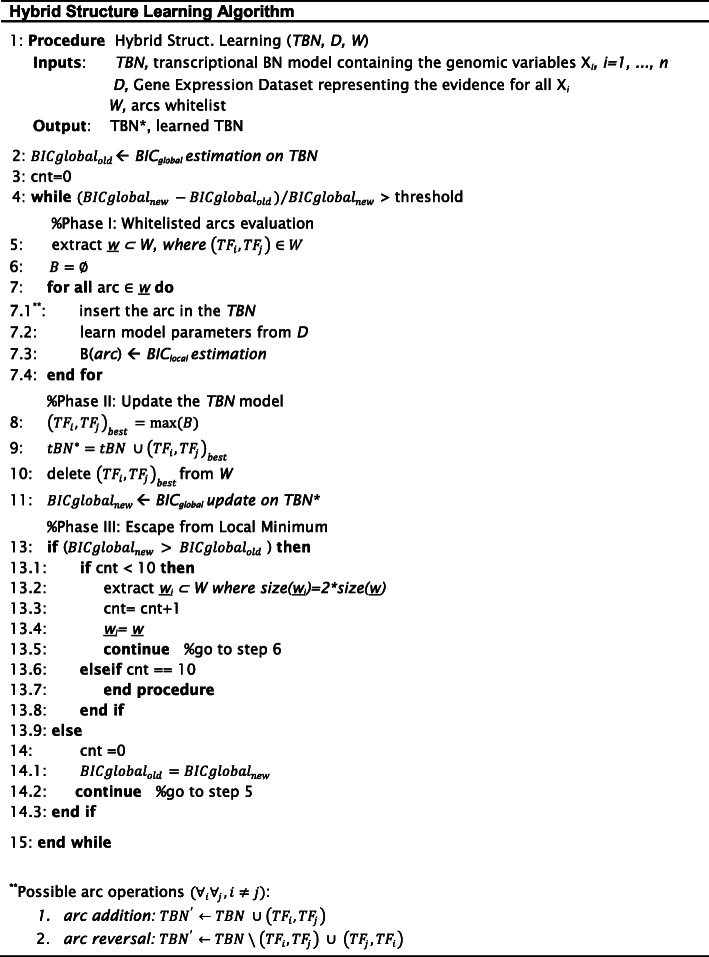
Pseudo code of the hybrid algorithm for learning a transcriptional BN structure

The hybrid algorithm developed in the current study proposes a heuristic search over the space of all possible structures derived from the whitelist, which encloses the informative structure priors concerning the TF-TF relations. All steps of the learning process are referenced below and are presented in Fig. 9, in which the pseudo code of the algorithm, implemented in Matlab language, is reported.

The learning procedure is designed for parallel computing and it has been tested both on a single multi-core machine (P7 CPU 4.0 GHz, 32 GB RAM) and on a high-performance computing environment, the University of Florida HiPerGator 2.0 cluster (52,000 cores, with 4GB RAM per core).

At each iteration, the algorithm randomly draws a set of arcs (*w*) (i.e. one hundred) from the whitelist to test them in the transcriptional BN (step 5). This sampling process is guided by correlation, which is exploited as an extraction probability associated to each whitelisted edge. The algorithm adds every sampled arc, one by one, to the BN model, learns the model parameters from gene expression (GE) data, and evaluates the newly obtained BN using the *Bayesian Information Criterion* (BIC) scoring metric (steps 6–7). Exploiting the decomposability property of this scoring function [99], the score of a network (G) given the data (D) can be written as the sum of scores of individual variables, where the score of each variable is calculated considering only the variable and its parents, as reported in Eq. (3).

3 $$ Score\left(G\ |\ D\right)=\sum \limits_{i=1}^{\mathrm{n}} FamScore\left({X}_i\left|{pa}^G\left({X}_i\right)\right|D\right) $$

In particular, since the TBN distribution is assumed to be jointly multivariate Gaussian, the BIC score can be expressed in terms of the residual sum of squares (RSS)

4 $$ BIC= nlog\left( RSS/n\right)+ klog(n) $$

where *n* is the number of observations (the GE dataset size), and *k* is the number of parameters in the model.

Determining the optimal structure G* from a finite set of model structures requires selecting a model that maximizes Eq. (3), as

5 $$ {G}^{\ast }=\mathit{\arg}\underset{G}{\mathit{\max}} Score\left(G\ \right|\ D\Big) $$

Assuming that each variable of the BN model is linearly dependent upon its continuous parents, we consider the BN as the sum of all local models.

Thus, we modeled two BIC scores, a *local* one that is used to assess the local improvement in the network before and after a whitelisted arc addition, and a *global* one which represents the BN score computed as the sum of all BIC scores from local models, as shown in Eq. (6) and Eq. (7), respectively.

6 $$ {BIC}_{local}=\Delta  BIC={BIC}_{old}-{BIC}_{new}= nlog\left(\frac{RSS_{old}}{RSS_{new}}\right)-\Delta  k\ast \log (n) $$

7 $$ {BIC}_{global}=\sum \limits_{i=1}^m BIC $$

where *m* denotes the number of local models composing the transcriptional BN.

The second term in Eq. (6) is a penalty term that takes into account the edge changes; since many of the whitelisted arcs come from TRN regulatory loops, the algorithm can add a new arc between two nodes (*∆k* = 1) or reverse the directionality of an existing BN arc (*∆k* = 0), as illustrated in the box of Fig. 8.

Our learning scheme is designed for parallel computing, allowing to test all the arcs extracted from the whitelist simultaneously. The algorithm evaluates all the computed *BIC*_*local*_, selects as best model the solution that maximizes Eq. (6) (step 8), and then includes the corresponding arc into the model (step 9). The BN structure and its new score (*BICglobal*_*new*_) are updated, and the process moves forward (steps 9–11) until the stop criterion (defined at the step 4) is met. The algorithm ends its iterations when the new model score does not improve more than a fixed threshold compared to the score of the previous network (*BICglobal*_*old*_). This threshold is estimated as 10 ∗ (*BIC*_*global*_)^−1^ calculated on the initial TRN, given as input of the hybrid algorithm.

The learning procedure also provides a strategy to prevent the search phase from getting trapped in a local optimal network (steps 13–14). When the stop condition is verified, the algorithm tries to move out of this potential local minimum for 10 consecutive times, combining an increased arc sampling size (*w*_i_) (for instance, if dim(w) = 100, the dimension of *w*_i_ is doubled as dim (w_i_) = 200) with a correspondingly augmented proportion of arcs to test. We save the *BIC*_*global*_ computed on the model before starting this procedure as *BICglobal*_*old*_; if in any of these steps the *BIC*_*global*_ for the new solution (*BICglobal*_*new*_) is not better than the *BICglobal*_*old*_, at the last iteration, the algorithm stops, otherwise it accepts the new model structure and continues the search process. At the end of each algorithm run, the heuristic procedure returns as output a learned transcriptional BN.

### Consensus transcriptional BN definition

In order to obtain a final robust transcriptional model including the regulatory dependencies consistently found across all the learned TBNs, as pointed out in [100], we defined a “consensus approach” to identify structural consistencies among all the models gained from several runs of the learning algorithm. We determined a confidence threshold, as the minimum degree of confidence for an edge to be significantly accepted in a final Consensus Bayesian Network. For each learned TF-TF edge (*e*_*ij*_*),* we compute its strength (*w*_*ij*_) considering the BN models (*m*), in which this transcriptional relationship appeared, and their related scores (*BIC*_*global*_).

8 $$ {w}_{ij}=\sum \limits_{m=1}^n\left({BIC}_{global}(m)\right) $$

Edges with high confidence (significant edges present in more than half of the learned network structures, and in the best scenario, present in all the network structures) are strongly weighted and more likely to be included in the final consensus model.

The percentile distribution of the edge weights combined with the edge frequencies were used to rank all the considered arcs and to assess a confidence threshold, ensuring that the obtained transcriptional consensus BN is acyclic and fully connected.

### Competing methods

The peculiarities of our novel approach optimized for learning large-scale transcriptional BNs make finding other similar methods difficult, especially in the class of hybrid BN learning algorithms, which exploit prior knowledge, directed regulations, transcriptomics and epigenomics data. To evaluate the performance of our method, we selected the SAGA (Simulated Annealing with a Greedy Algorithm) algorithm [46], the only approach with some common grounds with our strategy, and ARACNe (Algorithm for the Reconstruction of Accurate Cellular Networks), which is the most widely used technique for regulatory network reconstruction from gene expression data [101]. Another tool for learning BN structures and estimating their parameters is the R package bnlearn [102], which however cannot be used for our purposes. Bnlearn implements the Max-Min Hill-Climbing as hybrid algorithm, but to reconstruct the network from GE data, it forces all the arcs of the structural prior, specified as a DAG within a *whitelist*, to be included in the final network, preventing the addition of any other extra transcriptional relationship. This constraint makes this approach not appropriate to handle transcriptional networks, and in particular our type of whitelist given the presence of regulatory loops.

SAGA is a hybrid Bayesian learning algorithm, implemented in the BANJO (Bayesian Network Inference with Java Objects) software [103], which combines Simulated Annealing with a greedy search, using Bayesian Dirichlet equivalence as a scoring metric to evaluate the generated network. It allows arc addition and reversal, and the possibility to specify a structural prior as well as a list of forbidden arcs that must not be added (blacklist) to the model. This method does not exploit an arcs whitelist strategy, but it infers the network structure from discretized gene expression data. BANJO ends its search when one of the termination criteria are met (i.e. fixed number of explored networks, search threshold time, maximum number of restarts reached), and returns as output the learned network with the best score.

ARACNe is an information-theoretic based approach that implements Data Processing Inequality on each connected gene triplet from the GE dataset, to remove the least significant edge in mutual-information (MI) relevant networks. For our test, we used the last version of this algorithm, ARACNe-AP [47], that works on reconstructing transcriptional networks taking as inputs a GE dataset and a predefined list of regulators (TFs). Its strategy consists of computing MI only for every TF/target pair, and reconstructing MI networks from bootstrapped GE samples. A consensus network is then generated from the significant edges detected across all bootstrap runs.

## Supplementary information

**Additional file 1.**

## Data Availability

All the datasets used and analyzed during the current study are publicy available in the following repositories. • Chronic Myeloid leukemia datasets: ENCODE ChIP-Seq K562 raw data https://www.encodeproject.org/search/?type=Experiment&status=released&biosample_ontology.term_name=K562&assay_title=ChIP-seq&biosample_ontology.classification=cell+line&files.file_type=bam&assay_title=TF+ChIP-seq Chronic myeloid leukemia Gene Expression data: GEO accessions: GSE1315, GSE47927, and GSE24739; ArrayExpress accessions: E-MTAB-2581, E-MEXP-480. • Yeast transcriptional datasets: SGD Project transcriptional interactions data https://yeastgenome.org/ YEASTRACT transcriptional interactions data http://yeastract.com/download.php • Hematopoietic transcriptional networks: The K562 DNaseI derived network is available in the supplementary data of Neph et al study [9]. The K562 TFs co-association network is available at http://encodenets.gersteinlab.org/ A Matlab implementation of our algorithm is available at https://github.com/esauta/TBN_learning

## Abbreviations

ATAC-Seq: Assay for Transposase-accessible Chromatin Sequencing
BIC: Bayesian Information Criterion
BN: Bayesian Network
ChIP-Seq: Chromatin ImmunoPrecipitation followed by Sequencing
DNase-Seq: DNase I hypersensitivity sites Sequencing
CML: Chronic Myeloid Leukemia
DAG: Directed Acyclic Graph
GE: Gene Expression
HM: Hierarchy metric
HSA: Hierchical Simulating Annealing algorithm
MR: Master Regulator
TBN: Transcriptional Bayesian Network
TF: Transcription Factor
TRN: Transcriptional Regulatory Network
