# Supplementary material for: A Bayesian data fusion based approach for learning genome-wide transcriptional regulatory networks
Source: BMC Bioinformatics. 2020 May 29;21:219. doi: 10.1186/s12859-020-3510-1 (PMC7257163; doi:10.1186/s12859-020-3510-1)
Supplement: Supplementary file 1 — Additional file 1. [file 12859_2020_3510_MOESM1_ESM.docx]

**Supplementary Figures**


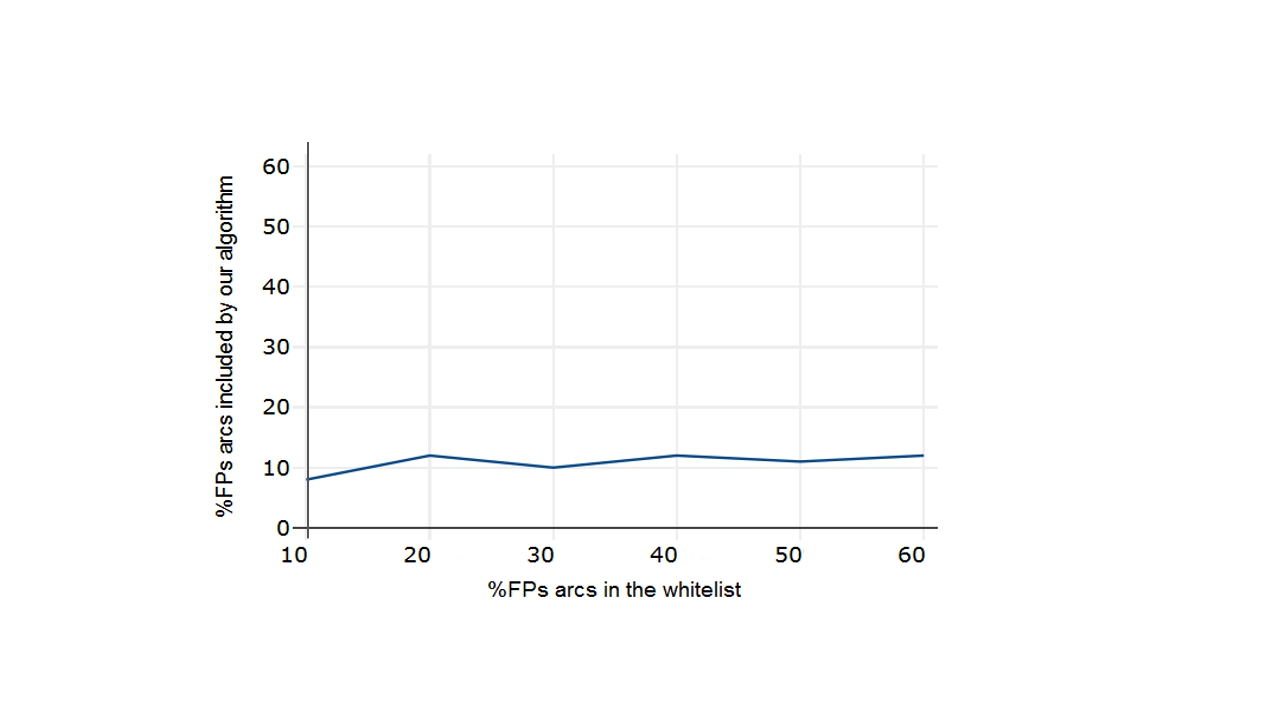


**Supplementary Fig. 1.** Robustness of our method to erroneous priors. For each considered FP rate, we reported the total rate of FPs enclosed in each whitelist compared to the percentage of FPs included in the final consensus BN. Despite the increasing FPs rate, the method shown robustness to wrong prior information that is intrinsic noisiness of experimental data source.

**
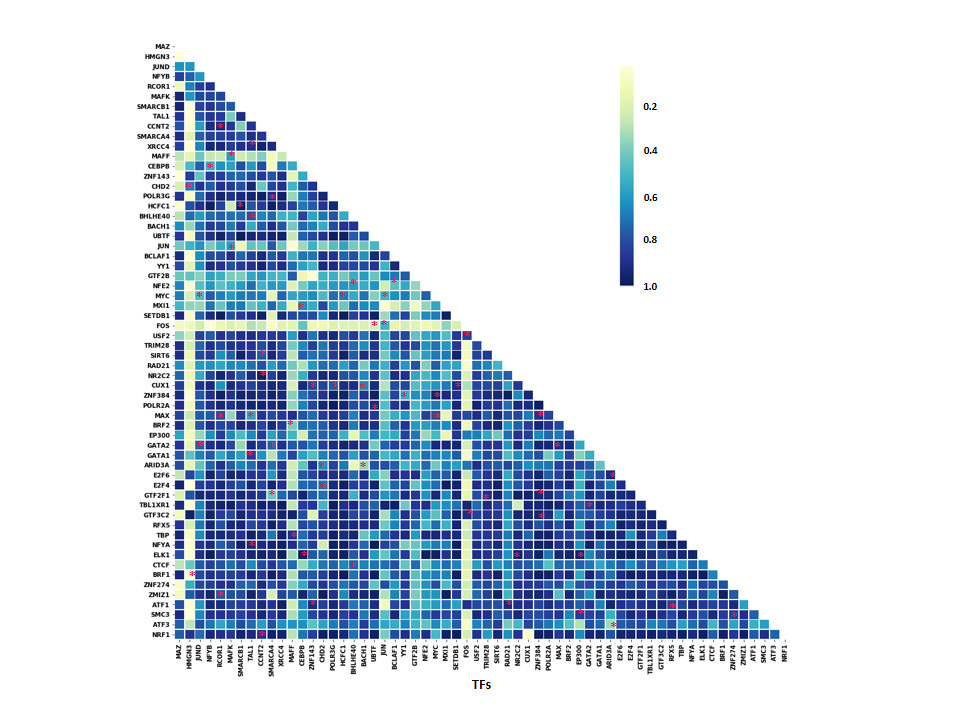
**

**Supplementary Fig. 2.** Correlation Heatmap. The Pearson Correlation was calculated between each pair of the 60 TFs, whose estimated values were tied to the related transcriptional edge as a sampling probability during the initial learning phase of the hybrid algorithm. As highlighted by red asterisks, all transcriptional dependencies included in the final consensus TBN have a high correlation.

**Major hematopoietic regulators subnetwork comparison**

The comparative analysis, performed on our ChIP-Seq derived transcriptional dependencies and the DNaseI-footprinted transcriptional network, was conducted considering all regulations driven by seven major and well-characterized hematopoietic transcription factors (TFs) TAL1/SCL, SP1/PU.1, ELF1, HES1, MYB, GATA1 and GATA2.

Since the genomic binding profile is available for three of the seven TFs, we examined all regulatory relationships from TAL1/SCL, GATA1 and GATA2, as main regulators, to all related TF interactors shared by both considered networks. As a result, we extracted a subnetwork composed of 18 TF nodes and 65 edges, in which all common transcriptional dependencies represents a 63% of regulatory overlap, as shown in Supplementary Fig. 3. Moreover, included in our prior, for the same core of 18 regulators, there are 162 transcriptional relationships of which 75% of these interactions are not present in the DNaseI derived network, but within our proposed integrative framework they can be exploited to depict a more comprehensive picture of the considered transcriptional context.


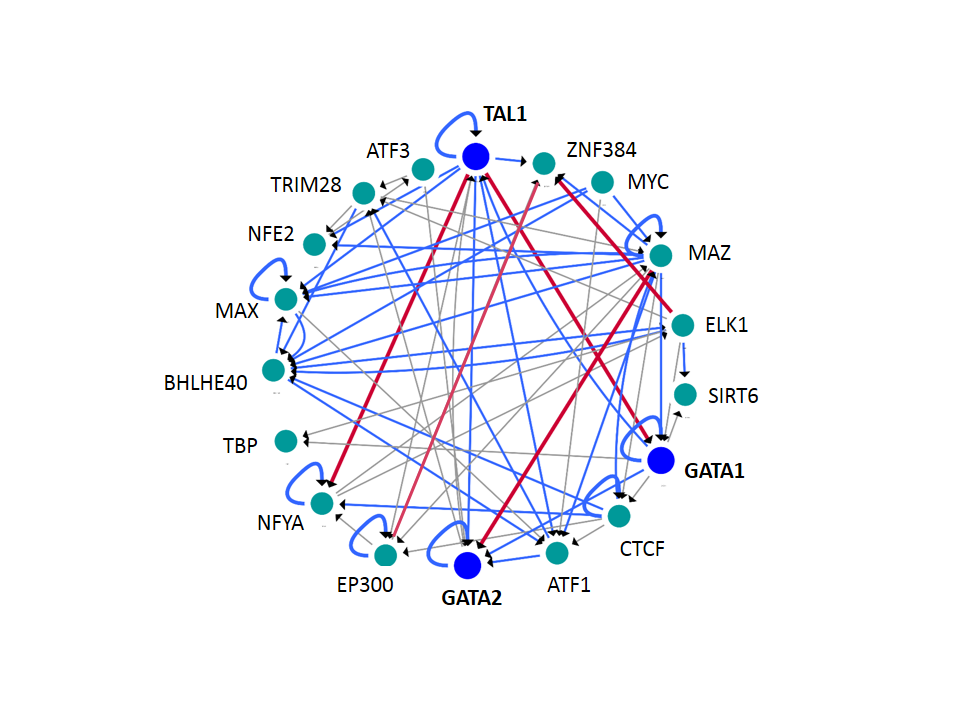


**Supplementary Fig. 3.** Three major hematopoietic TFs subnetwork comparison. Shown are all transcriptional regulations from the three main hematopoietic factors (blue colored nodes) to all common regulators (aquamarine colored nodes), for which the ChIP-seq binding profile is available in our prior. Shared transcriptional dependencies are highlighted in blue, and red arcs represent high confidence interactions embedded in the consensus TBN model.

**TF co-association based hematopoietic transcriptional network: Hierarchical comparison**

The structural comparison of transcriptional regulations was performed between a TF co-association network, whose interactions where obtained from a co-binding map (K562 ENCODE data) using a discriminative machine learning algorithm, and our transcriptional Bayesian model. To evaluate the similarity of transcriptional dependencies, we extracted from the TFs co-association network all interactions from factors in common with our TF set. The resulting subnetwork of 39 shared TF nodes included 464 edges, of which 93% of transcriptional regulations were found in our model. Given the high degree of similarity with our proposed regulatory diagram, we investigated if this transcriptional correspondence has an underlying common hierarchical organization. Since on the TF co-association model was applied a hierarchical simulating annealing algorithm (HSA) to order TFs in three discrete levels [1], we decided to perform the same procedure on the entire subnetwork, comparing the resulting order with the one estimated by the hierarchy height metric (HM), as used to derive our final consensus hierarchy. This measure was instead applied only on the edges fraction shared with the learned TBN to effectively evaluate the regulatory schema overlap.

The hierarchy generation algorithm was repeated for 1000 times (*k*=1,000), as suggested by the authors [2,3] and the number of hierarchical level fixed to three (*L*=3); the other parameters were set to default. The method determines the structure assigning each node to the level with highest frequency. The hierarchical structure predicted by the two methods is almost in complete agreement, reaching ~90% of shared regulatory positions. All master regulators were correctly placed, the little difference was between some factors of the middle and bottom levels, due to the small discrepancy of the considered edges. The preserved hierarchy highlights the robustness of learned transcriptional dependencies. The resulting assignments from the two measures are presented in Supplementary Figure 4, with the related number of matched TFs for each hierarchy level, and they are also reported in Supplementary Table 1.


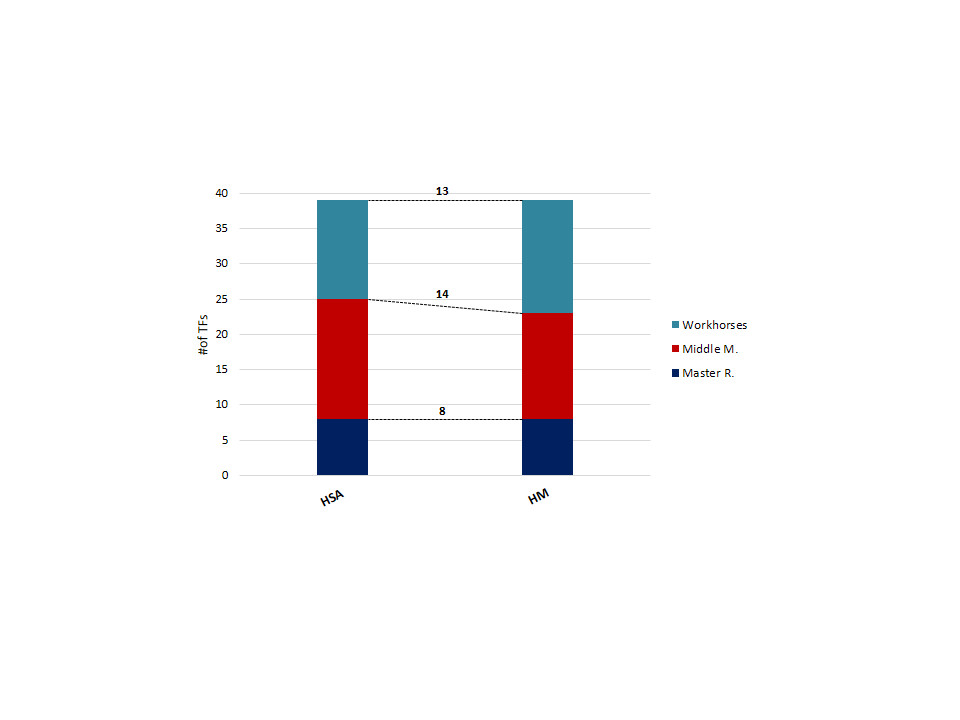


**Supplementary Fig. 4.** Hierarchical distributions inferred by HSA algorithm and HM measure. Highlighted between bars are the number of TFs predicted in the same hierarchical level by the two methods.

**Supplementary Table 1**

Summary of all assigned levels for each considered TF underlying hierarchical distributions predicted by hierarchical simulating annealing algorithm (HSA) and hierarchy height metric (HM). The following numeration represents (1) for the bottom layer, (2) and (3) for the middle and top layers, respectively.

| **TF** | **HSA**  **predicted level** | **HM**  **predicted level** |
| --- | --- | --- |
| YY1 | 2 | 2 |
| JUND | 1 | 1 |
| NFYB | 2 | 2 |
| NFYA | 1 | 1 |
| MAFK | 2 | 2 |
| TAL1 | 2 | 2 |
| CCNT2 | 3 | 3 |
| XRCC4 | 1 | 1 |
| CEBPB | 2 | 2 |
| CHD2 | 3 | 3 |
| SMC3 | 2 | 2 |
| FOS | 2 | 2 |
| JUN | 2 | 2 |
| BCLAF1 | 1 | 2 |
| GTF2B | 2 | 2 |
| NFE2 | 1 | 1 |
| MYC | 2 | 2 |
| SETDB1 | 2 | 1 |
| USF2 | 1 | 1 |
| HMGN3 | 3 | 3 |
| TRIM28 | 3 | 3 |
| SIRT6 | 1 | 1 |
| RAD21 | 3 | 3 |
| MXI1 | 2 | 2 |
| POLR2A | 1 | 1 |
| BRF2 | 1 | 1 |
| EP300 | 1 | 1 |
| GATA2 | 1 | 1 |
| GATA1 | 2 | 1 |
| E2F6 | 2 | 2 |
| E2F4 | 3 | 3 |
| GTF2F1 | 3 | 3 |
| MAX | 3 | 3 |
| TBP | 1 | 1 |
| CTCF | 2 | 2 |
| BRF1 | 2 | 1 |
| ZNF274 | 1 | 1 |
| ATF3 | 1 | 1 |
| NRF1 | 2 | 2 |

**Supplementary references**

[1] Gerstein MB, Kundaje A, Hariharan M, Landt SG, Yan K-K, Cheng C, et al. Architecture of the human regulatory network derived from ENCODE data. Nature 2012;489:91–100. https://doi.org/10.1038/nature11245.

[2] Cheng C, Andrews E, Yan K-K, Ung M, Wang D, Gerstein M. An approach for determining and measuring network hierarchy applied to comparing the phosphorylome and the regulome. Genome Biol 2015;16:63. https://doi.org/10.1186/s13059-015-0624-2.

[3] Ispolatov I, Maslov S. Detection of the dominant direction of information flow and feedback links in densely interconnected regulatory networks. BMC Bioinformatics 2008;9:424. https://doi.org/10.1186/1471-2105-9-424.
